# Anesthetic Management of a Patient With Parry-Romberg Syndrome: A Case Report

**DOI:** 10.7759/cureus.89051

**Published:** 2025-07-30

**Authors:** Reem F Bahakeem, Feras A Alotaibi, Abdulqader K Atiah, Majed A Basodan, Muwaffaq N Abutaha

**Affiliations:** 1 Medicine, Umm Al-Qura University, Makkah, SAU; 2 Anesthesiology, King Fahad General Hospital, Ministry of Health, Jeddah, SAU; 3 Anesthesiology, King Abdulaziz Hospital, Jeddah, SAU; 4 Anesthesiology, King Fahad Armed Forces Hospital, Jeddah, SAU

**Keywords:** advanced airway management, anesthetic case report, craniofacial deformity, difficult airway, hemifacial atrophy, nasal intubation, parry-romberg syndrome, pediatric anesthesia, video laryngoscopy

## Abstract

Parry-Romberg syndrome (PRS), also known as progressive hemifacial atrophy, is a rare condition characterized by unilateral degeneration of facial tissues. This case report details the anesthetic management of a 16-year-old female with PRS scheduled for elective dental surgery. The patient’s facial asymmetry and restricted mouth opening posed significant challenges for airway access, necessitating a customized anesthetic plan. Despite difficulties with visualization and nasal intubation due to anatomical limitations, the airway was successfully managed using video laryngoscopy and blind nasal intubation. The procedure proceeded without complications, and the patient had an uneventful recovery. This case highlights the importance of individualized planning in patients with complex craniofacial abnormalities.

## Introduction

Parry-Romberg syndrome (PRS) is a rare, progressive disorder characterized by unilateral atrophy of facial structures [1], often accompanied by neurological and ophthalmological involvement. The condition typically presents in childhood or early adulthood and can lead to both functional limitations and cosmetic disfigurement. Although the exact etiology remains uncertain, autoimmune, neurogenic, and infectious mechanisms have been suggested. Craniofacial asymmetry, soft tissue loss, and skeletal deformities associated with PRS can contribute to challenging airway management, making anesthesia particularly complex in these patients.

Given the risk of airway-related complications, anesthesiologists must undertake thorough preoperative assessments, formulate detailed anesthetic plans, and prepare multiple backup strategies. Additionally, patients may present with neurological manifestations such as trigeminal neuralgia or seizures, which can further complicate anesthetic care.

This case report presents the anesthetic considerations and intraoperative airway challenges encountered in a 16-year-old female with PRS undergoing elective dental surgery under general anesthesia.

## Case presentation

A 16-year-old female with a confirmed diagnosis of PRS presented for elective dental surgery. She exhibited progressive left-sided hemifacial atrophy, resulting in both aesthetic concerns and functional difficulties, including episodes of trigeminal neuralgia. Her mental status was unremarkable.

Preoperative assessment

Physical examination revealed marked facial asymmetry, including mandibular retrusion and dental misalignment. Neurological assessment showed left-sided ptosis and restricted cervical mobility, particularly on the affected side. The patient’s BMI was 15.4 kg/m², classifying her as underweight.

Airway assessment indicated several predictors of a difficult intubation. She had a Mallampati score of Class III, a thyromental distance of less than 6 cm, and restricted mouth opening limited to two fingers. Oropharyngeal examination revealed atrophy of both the tongue and uvula.

**Figure 1:**
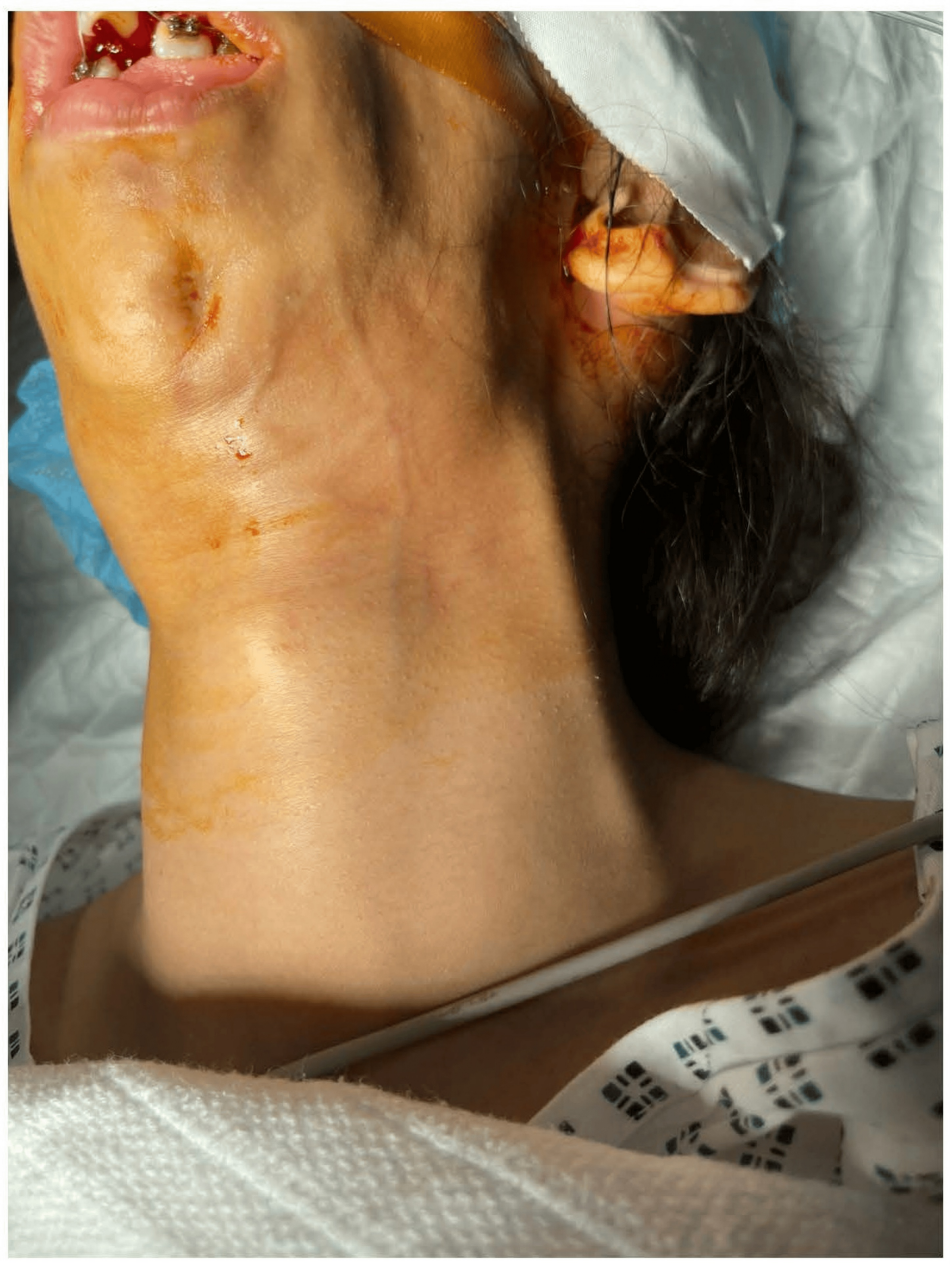
Clinical presentation of PRS showing hemifacial atrophy PRS, Parry-Romberg syndrome

Figure 1 shows a clinical photograph of the patient with left-sided hemifacial atrophy consistent with PRS. Notable features included mandibular retrusion, soft tissue wasting, a flattened nasolabial fold, and mild ptosis of the left eyelid. These asymmetrical findings contributed to anticipated challenges in airway management.

Baseline vital signs were within normal limits: a heart rate of 88 bpm, a respiratory rate of 18 breaths per minute, and a blood pressure of 110/70 mmHg. Laboratory investigations revealed a hemoglobin level of 10.5 g/dL (reference range: 12-16 g/dL for females) with normal electrolyte values. Imaging studies showed no additional structural abnormalities.

Anesthetic management

Given the patient’s complex craniofacial anatomy and anticipated airway difficulties, a comprehensive anesthetic plan was developed. A difficult airway algorithm was implemented, which included preparation of a laryngeal mask airway, multiple intubating stylets, various sizes of endotracheal tubes, and standby surgical tracheostomy. An ENT surgeon was on call and available throughout the procedure in the event of airway failure.

Preoperative optimization included intravenous fluid administration and anxiolysis with midazolam. The decision to administer midazolam was made following multidisciplinary discussion, weighing the patient’s significant procedural anxiety against the potential airway-related risks. A minimal dose was used, with continuous monitoring of ventilation and consciousness. Ventilation was closely monitored using capnography (ETCO₂), pulse oximetry (SpO₂), and clinical observation for any signs of respiratory compromise.

Advanced airway management tools, including a McGrath video laryngoscope, were prepared in anticipation of intubation challenges.

Intraoperative management

The patient was transferred to the operating room, where standard monitoring was established, including continuous ECG, pulse oximetry, and noninvasive blood pressure monitoring.

In light of the intubation requirements, nasal intubation was performed using a nasal RAE tube. This decision was influenced by the unavailability of an appropriately sized armored tube, which limited the options for tube selection. The use of a fiberoptic laryngoscope or a bougie through the nasal RAE tube posed significant technical challenges, leaving the McGrath video laryngoscope as the only viable alternative. Further complicating the procedure was the fact that only one nostril was available, as the other was affected by facial deformity.

The use of neuromuscular blockade during induction was a matter of discussion due to the anticipated difficulty with intubation. A consultant anesthesiologist recommended administering a high dose of muscle relaxant after confirming that mask ventilation was achievable, as this approach could improve the chances of successful intubation.

General anesthesia was induced using intravenous agents: propofol 60 mg, fentanyl 60 mcg, and rocuronium 25 mg. Following induction, ventilation was confirmed to be easy, prompting the administration of the muscle relaxant.

An initial attempt at direct laryngoscopy was unsuccessful due to the patient’s challenging airway anatomy. A McGrath video laryngoscope was subsequently used; however, restricted mouth opening made visualization difficult. Maneuvering the endotracheal tube with Magill forceps was also challenging. Multiple attempts using the McGrath laryngoscope allowed visualization of only the upper portion of the epiglottis (Cormack-Lehane grade IV). Consequently, a blind nasal intubation approach was attempted, in which the tube was placed beneath the epiglottis and advanced blindly into the trachea.

Although blind nasal intubation is typically considered a last-resort technique due to its risk of trauma and lower success rates, it was employed in this case due to several limiting factors. The patient’s anxiety and limited cooperation rendered awake or endoscopic intubation unfeasible. Additionally, the absence of a suitable atomizer for topical anesthesia and the unavailability of retrograde intubation equipment further restricted alternative strategies. Therefore, blind nasal intubation was carried out under full preparation and continuous monitoring.

Ultimately, successful endotracheal intubation was achieved, with proper tube placement confirmed by capnography and bilateral breath sounds.

Maintenance and intraoperative monitoring

General anesthesia was maintained with sevoflurane, and additional analgesia was administered as needed. Hemodynamic parameters remained stable throughout the procedure. Estimated blood loss was approximately 200 mL, and the surgery proceeded without complications.

Although the patient exhibited some neurological findings, there were no clinical or radiological signs suggestive of increased intracranial pressure. Standard neuroprotective measures, including head elevation and normocapnic ventilation, were implemented as a precaution.

Postoperative management

The patient was extubated while fully awake and transferred to the post-anesthesia care unit for close monitoring. Postoperative analgesia was provided with intravenous paracetamol and morphine, ensuring adequate pain control. Recovery was uneventful, and the patient was discharged after 24 hours with appropriate follow-up instructions.

## Discussion

PRS, a rare form of progressive hemifacial atrophy, presents a spectrum of anesthetic challenges, particularly in airway management. Facial asymmetry, soft tissue degeneration, and skeletal involvement can unpredictably alter upper airway anatomy, complicating both mask ventilation and endotracheal intubation. Moreover, associated neurological manifestations may further complicate perioperative management [2,3].

This case illustrates the severe end of that clinical spectrum. Our patient, a 16-year-old girl with advanced left-sided hemifacial atrophy, exhibited multiple predictors of a difficult airway, including a restricted oral aperture (two-finger breadth), Mallampati class III, and a Cormack-Lehane grade IV on laryngoscopy. Despite the use of a McGrath video laryngoscope, glottic visualization remained suboptimal, necessitating blind nasal intubation with the aid of Magill forceps, a technique rarely documented in recent PRS cases.

In contrast, one report described a PRS patient with right-sided atrophy and a Mallampati class I airway who underwent uneventful nasal intubation with a Cormack-Lehane grade I view, underscoring how the severity and laterality of PRS can influence airway complexity [1]. Similarly, another case documented successful intubation using a Glidescope in a patient without airway difficulty, again highlighting the variability in clinical presentation [2].

Although fiberoptic nasal intubation has proven effective in comparable cases, our setting lacked a compatible fiberoptic scope for nasal RAE tubes, a limitation reflecting common equipment constraints in resource-limited environments. This technical barrier necessitated the use of blind nasal intubation, a method less favored in modern practice but still a valuable fallback strategy [3].

Another study presented three cases of PRS with difficult mask ventilation, though intubation was ultimately successful using standard airway adjuncts. However, those patients had more favorable anatomy, particularly in terms of oral opening, compared to ours [4]. Likewise, a separate report described video-assisted oral intubation in a patient with minimal craniofacial involvement, requiring neither Magill forceps nor blind techniques. These disparities reinforce the importance of anatomical severity and asymmetry in guiding airway management strategies [5].

Although awake fiberoptic intubation is often recommended in patients with significant facial asymmetry, it was not feasible in our case due to the patient’s age, procedural anxiety, and limited anatomical access. These factors precluded patient cooperation and the safe use of an awake approach, highlighting the need to tailor airway strategies to individual patient characteristics rather than rely solely on algorithmic protocols [6].

Across the literature, certain anatomical features, such as low BMI, restricted mouth opening, and high Cormack-Lehane grades, consistently emerge as predictors of difficult airway management. Our patient exhibited several of these high-risk features, emphasizing the importance of thorough preoperative evaluation, multidisciplinary planning, and having both advanced and fallback airway tools available.

This case adds a unique perspective to the limited literature on PRS anesthesia by documenting successful blind nasal intubation in a high-risk patient under significant anatomical and equipment constraints. It reinforces the importance of individualized, flexible, and context-sensitive airway planning for patients with rare craniofacial syndromes. Continued reporting of such cases is essential to help shape a more standardized, evidence-based approach to anesthetic management in PRS.

## Conclusions

This case highlights the complex anesthetic considerations involved in managing a patient with PRS undergoing elective surgery. A structured, individualized approach - including meticulous airway evaluation and the use of both advanced and alternative intubation techniques - was crucial for ensuring patient safety and successful perioperative management. Despite limitations in equipment and challenges with patient cooperation, blind nasal intubation was successfully performed with full preparation and monitoring. This experience underscores the importance of weighing the risks of premedication in patients with compromised airways and adapting management strategies based on real-time anatomical and logistical constraints. These findings support the need for both flexible clinical judgment and the development of standardized anesthetic protocols tailored to rare craniofacial conditions.
